# Community-based surveillance of malaria vector larval habitats: a baseline study in urban Dar es Salaam, Tanzania

**DOI:** 10.1186/1471-2458-6-154

**Published:** 2006-06-15

**Authors:** Michael J Vanek, Bryson Shoo, Deo Mtasiwa, Michael Kiama, Steven W Lindsay, Ulrike Fillinger, Khadija Kannady, Marcel Tanner, Gerry F Killeen

**Affiliations:** 1 Swiss Tropical Institute, P.O. Box, 4002 Basel, Switzerland; 2 Ministry of Regional Administration and Local Government, Dar es Salaam, Tanzania; 3 School of Biological and Biomedical Sciences, South Road, Durham DH1 3LE, UK; 4 Ifakara Health Research and Development Centre, P.O. Box 53, Ifakara, Kilombero, Morogoro, United Republic of Tanzania

## Abstract

**Background:**

As the population of Africa rapidly urbanizes it may be possible to protect large populations from malaria by controlling aquatic stages of mosquitoes. Here we present a baseline evaluation of the ability of community members to detect mosquito larval habitats with minimal training and supervision in the first weeks of an operational urban malaria control program.

**Methods:**

The Urban Malaria Control Programme of Dar es Salaam recruited and provided preliminary training to teams of Community-Owned Resource Persons (CORPs) who performed weekly surveys of mosquito breeding sites. Two trained mosquito biologists accompanied each of these teams for one week and evaluated the sensitivity of this system for detecting potential *Anopheles *habitats.

**Results:**

Overall, 42.4% of 986 habitats surveyed by an inspection team had previously been identified by CORPs. Agricultural habitats were detected less often than other habitats (30.8% detected, Odds Ratio [95%CI] = 0.46 [0.29–0.73], P = 0.001). Non-agricultural artificial habitats were less suitable than other habitats (29.3% occupancy, OR = 0.69 [0.46–1.03], P = 0.066) but still constituted 45% (169/289) of occupied habitats because of their abundance (51 % of all habitats).

**Conclusion:**

The levels of coverage achieved by modestly trained and supported CORPs at the start of the Dar es Salaam UMCP were insufficient to enable effective suppression of malaria transmission through larval control. Further operational research is required to develop surveillance systems that are practical, affordable, effective and acceptable so that community-based integrated vector management can be implemented in cities across Africa.

## Background

With the prospect of more than half the African population living in urban areas by the year 2030, it is anticipated that the challenge and opportunity for tackling malaria burden in urban areas will also grow [1-3]. Compared to rural settings, malaria in urban Africa is generally characterized by lower intensities and more focal distribution of transmission, resulting in weaker immunity in the afflicted population and distribution of disease burden across older age groups [2,3]. Compared to rural settings, urban areas usually offer more malaria control options because relatively good transport, communication, educational and health infrastructure is available to large populations in small geographic areas. Through the relatively easy access to most urban area breeding sites, interventions such as environmental control and larvicide application may be cost-effective [2,3] but remain to be rigorously evaluated in the modern African context [4-6]. All previously documented successes of larval control against African malaria vectors have depended on rigorous surveillance for aquatic stage mosquitoes [7] to enable wholesale suppression [8] and even elimination [9,10]. To be sustainable in the context of African cities today, integrated vector management needs to be implemented through community-based systems using simple tools that are appropriately tailored to the enormous reservoir of affordable labor that is available *in situ *[11]. Operational research is required to develop surveillance systems that are practical, affordable, effective and acceptable but such investment could enable community-based integrated vector management in a variety of settings across Africa [11]. Here we describe a baseline evaluation of locally-recruited community-based personnel, in terms of their ability to detect potential *Anopheles *breeding sites, in Dar es Salaam, Tanzania.

The main vectors of malaria in the area of Dar es Salaam are *Anopheles gambiae sensu stricto*, *A. arabiensis*, *A. funestus *and *A. merus*. *Plasmodium falciparum *is the most common malaria parasite, accounting for 90% of all cases [12]. The distribution of larval habitats and transmission patterns is highly heterogeneous across space and time in the city of Dar es Salaam [13,14]. The development cycle from egg to adult for the main target species in the city, namely *Anopheles gambiae sensu lato*, can be completed in less than 7 days under optimal conditions [15-17]. Furthermore, *An.gambiae *often breeds in small aquatic habitats [15,16] which may escape detection by remote sensing techniques such as aerial photography and need to be verified on the ground. Risk maps therefore need to be updated on a regular basis to keep up with the rapidly changing field situation. A recently initiated Urban Malaria Control Programme (UMCP) in Dar es Salaam attempts to address this challenge by delegating responsibility for routine mosquito surveillance to modestly paid community members, known as Community-Owned Resource Persons (CORPs) [11]. The goal of this programme is to show that appropriately trained and managed CORPs can effectively survey and alleviate malaria burden in their locality. This study was carried out in order to evaluate the sensitivity of these CORPS teams under baseline conditions before the development of improved training, supervision and support systems by the UMCP.

## Methods

### Study site

**Figure 1:**
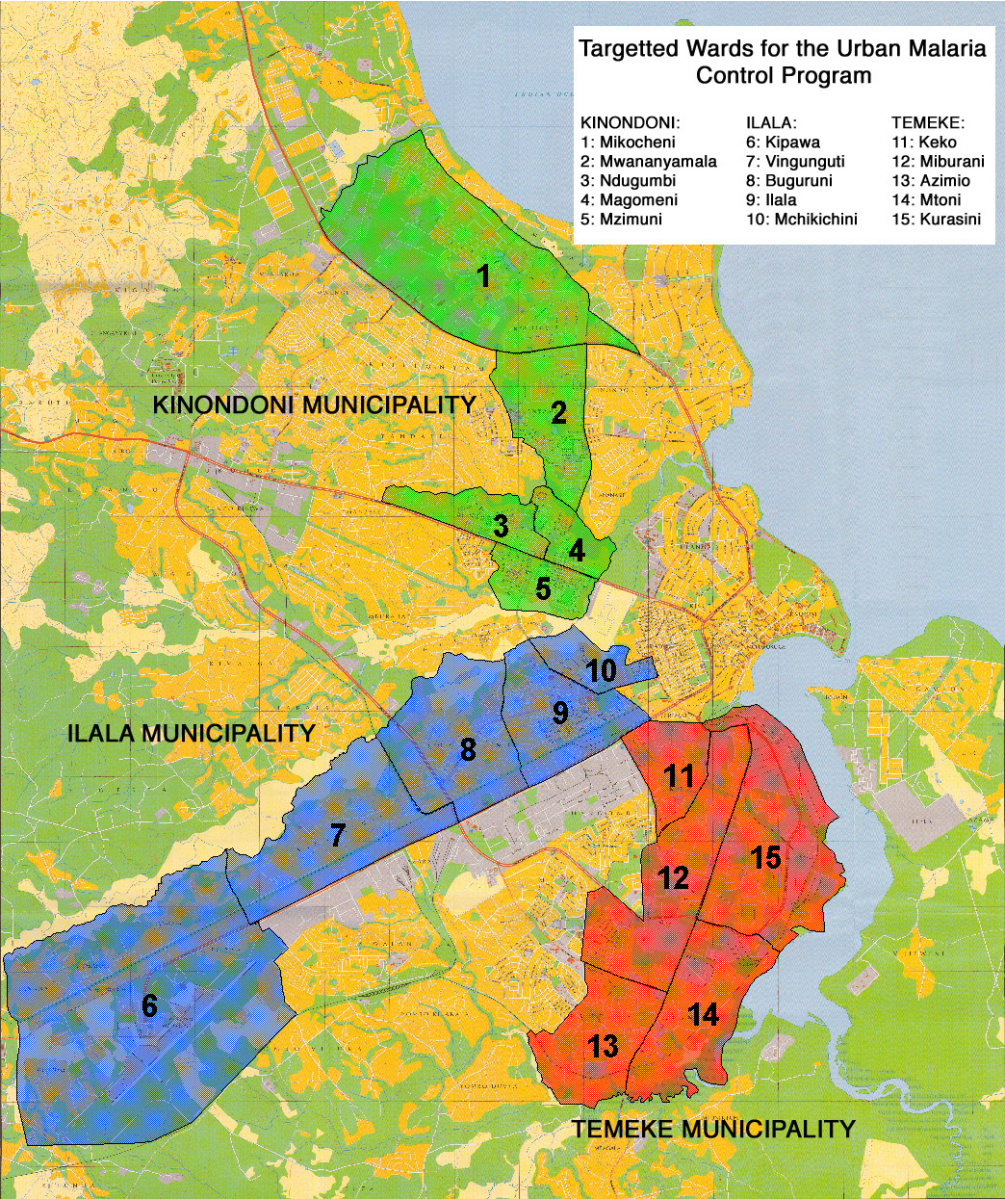
Wards included in the study area of the Dar es Salaam Urban Malaria Control Programme.

The study was conducted in Dar es Salaam, Tanzania's biggest and economically most important city with 2.5 million inhabitants and a total area of 1400 km^2 ^[12,18]. The city is divided into 3 municipalities, namely Ilala, Kinondoni and Temeke and each of these municipalities is further divided into wards. The 15 wards included in the Dar es Salaam UMCP (Figure 1) were chosen so that they encompass as wide a variety of malariological situations as possible and cover an area of 55 km^2 ^with wards ranging in size from 0.96 to 15 km^2^. In 2002, 611,871 people lived within this area [18]. With a high groundwater table and low elevation, the city is dotted with marshes, swamps, ponds and lakes that, combined with disturbances resulting from human activities, provide excellent breeding sites for mosquitoes [12,13].

The study was conducted from March to June 2004, during the main rainy season. Rainfall recorded at Dar es Salaam airport for this period and for the year as a whole were 494 and 913 mm, respectively. These were equivalent to 74 and 78% of the means for the previous decade, representing a slightly but not exceptionally dry year.

### Estimating the effectiveness of the Community Owned Resource Persons (CORPs)

Approximately 73 CORPs were employed at any given time during the study and these were each assigned defined areas based on local knowledge of habitat abundance, difficulty of terrain and geographic scale of the neighborhoods they were based in. All CORPs were nominated by Street Health Committees and received minimal remuneration as part-time workers through an employment system developed by the municipal councils of Dar es Salaam for sundry small scale maintenance tasks such as road cleaning. No larval control interventions were applied in any part of the study area during the period described.

Two trained mosquito biologists (MJV & BS) inspected each ward for one week and joined the CORPs on their weekly surveys through the wards they were responsible for. The study was initiated in Ilala municipality because a pilot version of these community-based surveys had been implemented there already in 2002. These two authors accompanied the CORPs in the 5 wards of Ilala municipality on their routine surveys and recorded joint observations with them without distinguishing between those found by the CORPs and by the authors. This pilot survey allowed adaptation of the protocol as follows before we started our research in the remaining 10 wards of Kinondoni and Temeke municipalities. Initially, the CORPS led the inspecting team to potential *Anopheles *larval habitats they had already recorded and described during previous surveys. Additional larval habitats identified by the inspecting team that had not been detected by the CORPs were also recorded and described. To quantify the sensitivity of the surveillance system and estimate differences in sensitivity between different habitats types, we applied binary logistic regression, with the proportion of all habitats found that were identified by the CORPs as the outcome variable.

### Habitat recording procedure

Every water body found in a ward was geo-referenced with a GPS-unit, described by using a standardized form and classified as one of the following habitat types: 1) Pits, holes and trenches associated with construction, 2)drains and ditches, 3)freshwater swamps with tall vegetation, 4) mangrove swamps, 5) marshes with short vegetation, 6) agricultural ridge and furrow systems (known locally as 'matuta' [13]), 7) puddles, 8) rice fields, 9) other forms of agriculture, 10) river and stream beds, 11) seepages and springs, 12) tyre tracks, 13) water storage containers and 14) any other type (See Table 1). The habitat type 'other' comprised mostly of wells, old tyres and depressions in the ground). Regrouping the 13 different habitat types into 3 major categories and treating "others" as a fourth, provided a better understanding of important breeding sites: Naturally formed habitats, such as 'freshwater swamp', 'mangrove swamp', 'marsh', 'river bed' and 'seepage' made up category number 1, the 'natural habitats'. Habitats, that belong to the agricultural sector, like crops grown in ridge and furrow systems, rice fields and "other agriculture", formed category number 2, namely 'agricultural artificial habitats'. All other artificial habitats, such as 'construction', 'drain', 'puddle', tyre track' and 'water storage tank' were included in category number 3, named 'non-agricultural artificial habitats'.

The presence of larvae was determined by dipping potential breeding sites [19]. Up to 10 dips were taken with a standard white 350 ml dipper and larvae density was classified as: No larvae (none of the dips contained larvae), larvae at low densities (an average of one larva per dip or less) and larvae at high density (an average of more than one larva per dip) as previously described [13]. *Anopheles *and culicine mosquitoes were differentiated macroscopically in the dipper. The distinctive feature applied was the floating habit of the mosquito larvae [20] and no further differentiation to species level was attempted. Morphological differentiation of pupae from different genera is very difficult and impracticable under field conditions in an operational malaria control program [13,21]. Pupae were therefore not differentiated between *Anopheles *and other genera.

### Ethics

All our work during this study was on biological and geographical material and did not involve human subjects. Research clearance was obtained from the Medical Research Coordination Committee of the National Institute of Medical Research in Tanzania (NIMR/HQ/R.8a/Vol. IX/279) and the Tanzanian Commission of Science and Technology (No. 2004-69-MFS-2004-24). This manuscript has been published with kind permission of the Director of the National Institute for Medical Research of the United Republic of Tanzania.

## Results

Out of the 986 potential habitats recorded by the inspection team in Kinondoni and Temeke municipalities, the CORPs had already detected almost half of these (Table 1). Logistic regression revealed significant differences between the sensitivity of detection of different habitat types (P < 0.001) and categories (P = 0.002). Although none of the individual habitat types were significantly more or less likely to be detected, a number of non-agricultural artificial habitats, as well as natural seepages appeared to be more readily detected. In contrast, agricultural sites other than ridge-and-furrow agriculture or rice were less likely to be detected. Overall, agricultural habitats and sundry uncategorized habitats were detected with approximately half the sensitivity of other artificial habitats and natural habitats. Indeed less than a third of these were previously detected by the CORPs surveys. Natural and agricultural habitats accounted for only 12.9 and 17.4% of all habitats, respectively, while non-agricultural artificial habitats constituted 51.0% of the total and sundry other habitats made up the remaining 17.3%.

Out of all the surveyed habitats, almost a third were occupied by *Anopheles *larvae. While this was significantly lower (P < 0.001 by χ^2 ^test) than previously reported for the same area in the dry season [13], it nevertheless indicates abundant proliferation of malaria vectors in urban Dar es Salaam during the rainy season. Habitat types and categories also varied significantly in terms of their occupancy by *Anopheles *larvae (P < 0.001 for both by logistic regression). Although part of this heterogeneity undoubtedly reflects differences in the sensitivity of sampling such diverse habitat types and sizes, several of the observed differences are opposite in magnitude to that expected based on the properties of the habitat, indicating genuine biological differences. Consistent with previous reports in Dar es Salaam [13] and the ecology of *An. gambiae *generally [15,16], swamps and artificial containers were relatively unsuitable habitats even though these habitats are expected to be relatively easy to sample and detect larvae within. Otherwise all habitat types proved highly suitable for *Anopheles*: Marshes, seepage, furrows associated with crops, rice fields, puddles and tyre tracks proved particularly receptive and suitable for larval growth of *Anopheles *(Table 2). Overall, non-agricultural artificial habitats proved substantially less suitable than natural and agricultural habitats but still constituted 45% (129/289) of all *Anopheles *occupied habitats because they comprised almost half the number of observed habitats. Sundry other habitats were even less suitable, having one third of the occupancy of natural habitats and constituting less than a fifth of all habitats.

## Discussion and conclusion

While this study was never intended as a comprehensive survey of larval habitats in Dar es Salaam, the relatively small areas and limited number of habitats sampled provide an indication of the potential and limitations of modestly trained and supported CORPs for surveillance of *Anopheles *proliferation in African cities. Our baseline results from Dar es Salaam suggest that man-made larval habits constitute the bulk of vector sources in this urban context. While CORPs surveys at this early stage of the Dar es Salaam UMCP detected less than half of almost all habitat types, they proved particularly poor at surveying agricultural habitats, perhaps because of the difficult nature of the often swampy terrain they were typically found in. This is of particular concern because these can be quite large areas, capable of producing large numbers of *Anopheles *and would be important targets for larval control.

The question of how much coverage with larviciding will be required to achieve worthwhile impact, and how best to achieve these levels under programmatic conditions, has been the focus of recent debate [22-24]. In principal, operational personnel would not necessarily need to achieve full coverage of all habitats if their productivity is highly heterogeneous and those few habitats responsible for the bulk of adult mosquitoes could be reliably identified [22-24]. Encouraging recent work using more intensive approaches for sampling pupae and emergent adults indicates quite specific land cover [25] and habitat types [26,27] could be targeted within relatively small localities. The potential for combining these ground-based sampling approaches with modern remote sensing technologies and hydrological models [28-30] may allow useful guidance for targeting in specific localities. However, scaling up such approaches to programmatic scale across districts, regions and countries remains unproven. Furthermore, current methods for detecting mosquito pupae or emergent adults are too intensive to apply routinely on large scales and the generalizability of such analyses remain to be determined. Even if reliable targeting criteria could be identified, the successful application of such elegant but technically complex criteria by community-based personnel in the most disadvantaged countries in the world is difficult to envisage in the near future. We therefore conclude that until reliable, practical and affordable means for targeting particular habitat locations or types are developed, comprehensive coverage will be essential if larval control is to achieve substantial reductions of malaria transmission and disease burden [22]. We propose that the proven individual protection afforded by an insecticide-treated net (ITN) [31], the front line malaria prevention tool in Africa today [32], represents an excellent gold standard with which larval control should be compared and contrasted. Despite the massive transmission challenge experienced by most citizens of sub-Saharan Africa [33,34], properly maintained ITNs consistently achieve cost-effectiveness equivalent to childhood vaccination [35] by preventing approximately 70% of exposure to infectious mosquito bites [36]. Assuming a linear relationship between coverage with larval control and reduction of malaria transmission [5,22], we therefore suggest that to achieve comparable impact at justifiable cost, larval control should aim for coverage levels of 70% or more at a total programme cost of 1 US$ or less per person protected per year. Both of these targets have been accomplished by recent small-scale efficacy trials [37] so a key challenge is to translate this efficacy into operational effectiveness through large-scale community-based programmes [11,22].

Community-based control strategies for malaria prevention have received considerable recent attention as a means to finally deliver the elusive goal of integrated vector management in impoverished African nations [11,26,38,39]. However, the community-based surveillance teams in Dar es Salaam evaluated here did not achieve 70% coverage for any habitat type or category and the overall coverage observed falls far short of this target. Thus, the sensitivity of larval surveillance at this early stage of the programme was clearly not sufficient to monitor and manage the wholesale suppression strategies that have underpinned previous successes of larval control [4-10]. If such community-based personnel are to achieve high levels of coverage in terms of detecting and killing *Anopheles *larvae, improved training, supervision and support tools will need to be developed that are practical and affordable in the poorest countries in the world. Investment in the development such broadly applicable tools could enable community-based integrated vector management in a variety of settings across Africa [11].

## Competing interests

The programme evaluated in this manuscript is partially supported by Valent Biosciences Corporation, a commercial manufacturer of microbial larvicides. Also, a substantial portion of the current salary and research support for the investigators depends on the achievement of documented suppression of malaria transmission and infection risk by this programme through systematic larviciding.

## Authors' contributions

MJV designed and implemented the study, analyzed the data and drafted the manuscript. BS, DM, MGK and KK were involved in designing and implementation the study. UF, SWL, MT and GFK participated in the study design, data analysis and drafting of the manuscript. All authors read and approved the final manuscript.

## Pre-publication history

The pre-publication history for this paper can be accessed here:



## Figures and Tables

**Table 1:** Detection coverage of different mosquito larval habitat categories and types by community-owned resource persons (CORPs).

Habitat Category	Habitat Type	Number detected			Coverage by CORPs surveys*		
		CORPs	Inspectors	Total	Detected by CORPS (%)	OR	P
*Natural Habitats*							
	Freshwater Swamp	10	15	25	40.0	1.00**	NA**
	Mangrove Swamp	1	2	3	33.3	NE	NE
	Marsh	21	29	50	42.0	1.09 [0.409–2.887]	0.868
	River bed	1	1	2	50.0	NE	NE
	Seepage	36	25	61	59.0	2.16 [0.836–5.58]	0.112
	*Subtotal:*	*69*	*72*	*141*	*48.9*	1.00**	NA**
*Agricultural artificial habitats*							
	Ridge and furrow	20	47	67	29.9	0.64 [0.25–1.66]	0.357
	Rice paddy	16	19	35	45.7	1.26 [0.45–3.57]	0.660
	Other agriculture	17	53	70	24.3	0.48 [0.18–1.27]	0.139
	***Subtotal:***	***53***	***119***	***172***	***30.8***	***0.46 [0.29–0.73]***	***0.001***
*Non-Agricultural artificial habitats*							
	Construction	38	43	81	46.9	1.33 [0.53–3.30]	0.544
	Drain	115	108	223	51.6	1.60 [0.69–3.71]	0.276
	Puddle	43	32	75	57.3	2.02 [0.80–5.07]	0.136
	Tyre track	28	18	46	60.9	2.33 [0.86–6.31]	0.095
	Water storage container	5	11	16	31.3	0.68 [0.18–2.56]	0.571
	*Subtotal:*	*229*	*212*	*441*	*51.9*	*1.11 [0.76–1.63]*	*0.587*
Uncategorized							
	Other	67	165	232	28.9	0.61 [0.26–1.43]	0.252
	***Subtotal***	***67***	***165***	***232***	***28.9***	***0.42 [0.27–0.65]***	***<0.001***
*Total*		*418*	*568*	*986*	*42.4*	*NA*	*NA*

* The proportion of all habitats found by the combined observations of the CORPs and the inspecting team that had previously been found by the CORPs. Odds ratios (OR) and P values for the likelihood of detection by the CORPs were determined by logistic regression, treating habitat category or type as a determinant of detection.

** Reference group

NE Not estimated: excluded from logistic regression analysis because of insufficient observations.

NA Not Applicable

**Table 2:** Occupancy of different mosquito habitat categories and types by *Anopheles *larvae.

Habitat Category	Habitat Type	*Anopheles *occupancy*					
		Present	Absent	Total	Proportion occupied (%)	OR	P
*Natural Habitats*							
	Freshwater Swamp	2	23	25	8.0	1.00**	NA**
	Mangrove Swamp	1	2	3	33.3	NE	NE
	**Marsh**	**19**	**31**	**50**	**38.0**	**7.05 [1.49–33.3]**	**0.014**
	River bed	2	0	2	100.0	NE	NE
	**Seepage**	**29**	**32**	**61**	**47.5**	**10.4 [2.3–48.1]**	**0.003**
	*Subtotal:*	*53*	*88*	*141*	*37.6*	1.00**	NA**
*Agricultural artificial habitats*							
	**Ridge and furrow**	**35**	**32**	**67**	**52.2**	**12.6 [2.7–57.6]**	**0.001**
	**Rice paddy**	**14**	**21**	**35**	**40.0**	**7.67 [1.56–37.8]**	**0.012**
	Other agriculture	18	52	70	25.7	3.98 [0.85–18.6]	0.079
	*Subtotal:*	*67*	*105*	*172*	*39.0*	*1.06 [0.67–1.67]*	*0.805*
*Non-Agricultural artificial habitats*							
	Construction	19	61	81	23.8	3.58 [0.77–16.6]	0.103
	Drain	57	166	223	25.6	3.95 [0.90–17.3]	0.068
	**Puddle**	**36**	**39**	**75**	**48.0**	**10.6 [2.34–48.3]**	**0.002**
	**Tyre track**	**16**	**30**	**46**	**34.8**	**6.13 [1.28–29.4]**	**0.023**
	Water storage container	1	15	16	6.3	0.77 [0.06–9.22]	0.834
	*Subtotal:*	*129*	*311*	*441*	*29.3*	*0.69 [0.46–1.03]*	*0.066*
Uncategorized							
	Other	40	191	232	17.3	2.41 [0.55–10.6]	0.246
	***Subtotal***	***40***	***191***	***232***	***17.3***	***0.348 [0.215–0.563]***	***<0.001***
*Total*		*289*	*695*	*986*	*29.4*	*NA*	*NA*

* The proportion of all habitats found to contain *Anopheles *larvae. Odds ratios (OR) and P values for the likelihood of occupancy were determined by logistic regression, treating habitat category or type as a determinant of detection.

** Reference group

NE Not estimated: excluded from logistic regression analysis because of insufficient observations.

NA Not Applicable
